# Supplementary material for: A Likelihood Approach for Real-Time Calibration of Stochastic Compartmental Epidemic Models
Source: PLoS Comput Biol. 2017 Jan 17;13(1):e1005257. doi: 10.1371/journal.pcbi.1005257 (PMC5240920; doi:10.1371/journal.pcbi.1005257)
Supplement: S1 File — (TAR.GZ) [file pcbi.1005257.s014.tar.gz › HSPH_Online-SI-Revision/Additions/IPoi10/n100-extreme_noisy/n100-extreme_noisy_BENCH2_table0.pdf]

1	newly 3 weeks spec	7368.61	8273.78	{5231., 11 709.}	27.9451	0.112032	newly 3 weeks cumul	27 409.9	28 769.5	{20 401., 35 705.}	18.1854	0.0781784	newly 5 weeks spec	4368.49	5159.18	{2763., 8013.}	40.7547	0.150699	newly 5 weeks cumul	37 692.1	40 591.6	{27 855., 53 138.}	21.357	0.0891365	newly 54 weeks cumul	51 622.1	54 329.	{34 625., 74 765.}	25.6278	0.10822
2	newly 3 weeks spec	5965.	3938.98	{1835., 7420.}	44.1309	0.269163	newly 3 weeks cumul	20 994.	14 597.	{7345., 26 213.}	37.9128	0.226073	newly 5 weeks spec	3796.	2478.62	{1095., 5026.}	49.1223	0.300173	newly 5 weeks cumul	29 744.	20 211.5	{9803., 37 594.}	41.0405	0.246641	newly 50 weeks cumul	42 279.	28 280.7	{13 164., 54 304.}	44.5413	0.269081
3	newly 3 weeks spec	6513.	5823.93	{3241., 10 745.}	39.8251	0.195105	newly 3 weeks cumul	27 002.	22 665.	{14 359., 35 491.}	29.6463	0.1552	newly 5 weeks spec	3181.	3113.16	{1446., 5861.}	52.1132	0.228015	newly 5 weeks cumul	34 839.	30 111.4	{17 947., 51 656.}	34.0063	0.172726	newly 55 weeks cumul	41 209.	36 896.	{20 899., 64 034.}	37.8591	0.184557
4	newly 3 weeks spec	6774.	8893.31	{5176., 13 864.}	42.0394	0.142808	newly 3 weeks cumul	33 063.	37 060.2	{25 413., 49 402.}	24.1657	0.0991417	newly 5 weeks spec	2428.	3824.28	{1938., 6416.}	67.5028	0.18514	newly 5 weeks cumul	39 680.	46 805.7	{31 103., 65 630.}	28.0762	0.106434	newly 59 weeks cumul	42 886.	52 831.5	{33 969., 76 496.}	32.9418	0.119529
5	newly 3 weeks spec	8431.	7308.38	{4189., 11 628.}	29.9335	0.151399	newly 3 weeks cumul	31 964.	27 791.2	{18 253., 38 033.}	20.6185	0.10535	newly 5 weeks spec	4440.	3881.7	{2009., 6690.}	39.0577	0.191927	newly 5 weeks cumul	42 439.	37 052.9	{23 107., 54 718.}	24.4605	0.12377	newly 56 weeks cumul	51 326.	45 404.3	{27 273., 68 816.}	27.5711	0.136795
6	newly 3 weeks spec	7676.	4897.1	{2339., 7935.}	42.7818	0.261453	newly 3 weeks cumul	28 666.	19 256.9	{10 651., 29 685.}	35.9552	0.213945	newly 5 weeks spec	4357.	2673.29	{1223., 4537.}	48.7039	0.303672	newly 5 weeks cumul	38 952.	25 590.2	{13 615., 40 149.}	38.9882	0.234428	newly 54 weeks cumul	50 195.	32 151.8	{16 494., 50 970.}	42.0325	0.255578
7	newly 3 weeks spec	7326.	7278.29	{4376., 10 664.}	25.8169	0.117377	newly 3 weeks cumul	27 951.	25 169.4	{17 429., 32 264.}	17.0653	0.0851826	newly 5 weeks spec	3953.	4722.76	{2481., 7999.}	41.9256	0.154151	newly 5 weeks cumul	37 123.	35 848.5	{23 204., 48 242.}	20.7721	0.0973568	newly 52 weeks cumul	46 099.	49 990.7	{29 860., 75 154.}	28.9196	0.118876
8	newly 3 weeks spec	7809.	6192.02	{3422., 10 175.}	34.0187	0.187062	newly 3 weeks cumul	28 877.	23 399.3	{14 451., 34 740.}	28.078	0.152545	newly 5 weeks spec	4404.	3554.92	{1713., 6170.}	40.9816	0.22207	newly 5 weeks cumul	39 237.	31 687.4	{18 674., 48 905.}	30.1823	0.163313	newly 54 weeks cumul	50 550.	40 958.	{22 694., 64 755.}	33.6167	0.181518
9	newly 3 weeks spec	8491.	9199.73	{6542., 11 593.}	18.4183	0.0765676	newly 3 weeks cumul	32 010.	30 050.	{24 789., 33 977.}	9.29411	0.0489684	newly 5 weeks spec	4649.	6276.	{3778., 9747.}	41.2591	0.139173	newly 5 weeks cumul	43 099.	44 101.7	{33 471., 51 899.}	12.1435	0.0533905	newly 54 weeks cumul	54 004.	63 112.	{44 728., 82 195.}	22.9117	0.0876519
10	newly 3 weeks spec	3970.	6783.48	{3462., 10 664.}	75.9934	0.224004	newly 3 weeks cumul	22 883.	30 474.8	{17 904., 40 099.}	41.4169	0.14742	newly 5 weeks spec	1101.	2699.53	{1243., 4348.}	146.447	0.34579	newly 5 weeks cumul	26 005.	37 545.7	{21 254., 54 828.}	51.2262	0.171403	newly 58 weeks cumul	27 241.	41 653.8	{23 118., 61 376.}	58.6441	0.188136
11	newly 3 weeks spec	6532.	5394.59	{2980., 9775.}	34.8042	0.183097	newly 3 weeks cumul	23 257.	20 156.7	{12 095., 32 386.}	28.3874	0.144734	newly 5 weeks spec	3974.	3176.33	{1583., 6534.}	41.1219	0.220154	newly 5 weeks cumul	32 341.	27 496.9	{15 781., 47 346.}	31.263	0.161173	newly 52 weeks cumul	43 101.	36 269.7	{20 123., 66 425.}	34.1452	0.176845
12	newly 3 weeks spec	8024.	8448.06	{5811., 11 301.}	22.1535	0.0948178	newly 3 weeks cumul	31 244.	27 995.4	{21 025., 32 987.}	13.3249	0.0688536	newly 5 weeks spec	4154.	5782.25	{3392., 9041.}	49.1627	0.162088	newly 5 weeks cumul	41 376.	40 895.1	{30 579., 50 605.}	16.1446	0.0736554	newly 53 weeks cumul	50 863.	58 750.8	{39 983., 80 540.}	27.4462	0.10675
13	newly 3 weeks spec	7496.	5010.21	{2669., 8871.}	40.3913	0.242932	newly 3 weeks cumul	24 982.	18 120.4	{10 849., 26 887.}	31.869	0.181811	newly 5 weeks spec	4998.	3215.27	{1473., 5722.}	49.3172	0.300928	newly 5 weeks cumul	36 270.	25 394.4	{14 398., 42 376.}	36.1169	0.210803	newly 51 weeks cumul	54 152.	35 532.4	{18 886., 60 516.}	42.7855	0.257831
14	newly 3 weeks spec	5024.	8602.88	{5403., 12 352.}	72.3765	0.216906	newly 3 weeks cumul	30 056.	31 535.3	{23 282., 39 149.}	18.8902	0.0806674	newly 5 weeks spec	1153.	4628.75	{2566., 7142.}	301.453	0.56635	newly 5 weeks cumul	33 840.	42 614.5	{29 618., 55 830.}	32.3483	0.117627	newly 56 weeks cumul	34 818.	52 720.	{35 137., 71 405.}	53.451	0.171996
15	newly 3 weeks spec	5483.	9013.19	{5616., 12 551.}	66.6541	0.207773	newly 3 weeks cumul	26 035.	32 766.6	{23 700., 39 991.}	31.4504	0.118767	newly 5 weeks spec	1932.	5004.83	{2749., 7397.}	159.105	0.378695	newly 5 weeks cumul	31 301.	44 619.9	{30 521., 57 405.}	45.2971	0.155585	newly 56 weeks cumul	33 894.	55 964.	{36 147., 74 817.}	66.6064	0.208864
16	newly 3 weeks spec	6616.	6401.03	{4013., 9888.}	28.6351	0.127576	newly 3 weeks cumul	30 832.	25 607.6	{17 804., 36 463.}	24.0321	0.123866	newly 5 weeks spec	2584.	3249.05	{1924., 5351.}	42.2655	0.14294	newly 5 weeks cumul	37 486.	33 484.6	{22 505., 49 295.}	24.8534	0.120707	newly 56 weeks cumul	41 249.	40 489.	{26 491., 60 756.}	26.3613	0.116607
17	newly 3 weeks spec	6952.	6858.65	{4429., 9822.}	24.9856	0.119727	newly 3 weeks cumul	24 739.	23 776.5	{17 382., 30 291.}	18.1023	0.0942592	newly 5 weeks spec	4470.	4544.77	{2648., 6964.}	31.9043	0.138812	newly 5 weeks cumul	34 818.	33 977.3	{23 446., 45 583.}	21.4043	0.102828	newly 51 weeks cumul	48 456.	49 846.5	{31 877., 70 500.}	26.4928	0.114514
18	newly 3 weeks spec	7355.	8139.04	{5454., 10 367.}	23.3159	0.0954037	newly 3 weeks cumul	26 994.	26 030.1	{20 149., 30 603.}	14.0365	0.0673477	newly 5 weeks spec	4020.	6044.1	{3400., 9447.}	57.1155	0.179883	newly 5 weeks cumul	36 463.	39 210.2	{28 170., 47 514.}	18.2306	0.0762258	newly 52 weeks cumul	45 100.	60 934.2	{40 248., 86 553.}	40.3408	0.139509
19	newly 3 weeks spec	8310.	8117.51	{5068., 11 843.}	23.9836	0.108903	newly 3 weeks cumul	31 919.	28 675.9	{20 841., 35 134.}	15.2957	0.0752597	newly 5 weeks spec	4512.	4895.36	{2582., 8435.}	35.9396	0.143636	newly 5 weeks cumul	42 585.	40 006.3	{26 977., 52 994.}	18.7903	0.0889094	newly 54 weeks cumul	52 753.	52 677.4	{33 235., 77 813.}	23.7468	0.104769
20	newly 3 weeks spec	6547.	11 346.1	{6250., 17 403.}	75.8284	0.222373	newly 3 weeks cumul	38 691.	40 102.5	{29 099., 48 373.}	17.1432	0.0754361	newly 5 weeks spec	1542.	5540.98	{2375., 10 361.}	259.338	0.486922	newly 5 weeks cumul	43 499.	53 887.4	{35 767., 69 056.}	29.9959	0.111242	newly 59 weeks cumul	44 967.	62 933.9	{39 303., 86 644.}	44.3091	0.150163
21	newly 3 weeks spec	7119.	9520.15	{6184., 13 428.}	39.1445	0.136968	newly 3 weeks cumul	29 356.	32 920.5	{24 962., 39 052.}	18.9866	0.0766505	newly 5 weeks spec	3172.	5625.52	{3044., 9347.}	80.0126	0.219712	newly 5 weeks cumul	37 313.	46 050.7	{33 848., 57 100.}	27.824	0.104124	newly 56 weeks cumul	42 719.	58 881.3	{40 396., 81 498.}	41.125	0.140599
22	newly 3 weeks spec	6759.	7877.19	{4905., 11 826.}	33.3897	0.125273	newly 3 weeks cumul	29 716.	31 381.	{21 769., 42 660.}	22.0155	0.0938851	newly 5 weeks spec	2827.	3888.38	{2068., 6290.}	52.2878	0.160891	newly 5 weeks cumul	37 047.	40 882.2	{28 145., 57 434.}	25.3446	0.101575	newly 57 weeks cumul	41 620.	48 462.2	{31 829., 71 204.}	30.4254	0.114703
23	newly 3 weeks spec	6270.	5520.59	{3333., 8348.}	29.8707	0.148002	newly 3 weeks cumul	22 584.	19 748.9	{13 183., 28 213.}	24.6162	0.123614	newly 5 weeks spec	3912.	3630.33	{2042., 6078.}	37.1628	0.174395	newly 5 weeks cumul	31 596.	27 909.1	{17 924., 40 953.}	26.8668	0.133078	newly 50 weeks cumul	43 028.	40 351.2	{24 248., 62 548.}	31.5633	0.147277
24	newly 3 weeks spec	7567.	5933.62	{3971., 8285.}	27.2237	0.145893	newly 3 weeks cumul	27 322.	20 725.	{14 809., 27 004.}	24.5879	0.133832	newly 5 weeks spec	4888.	4021.35	{2460., 6397.}	32.5433	0.16846	newly 5 weeks cumul	38 440.	29 666.2	{20 843., 40 288.}	25.6793	0.138225	newly 50 weeks cumul	52 801.	44 048.6	{28 600., 64 707.}	27.9223	0.142825
25	newly 3 weeks spec	6782.	8428.57	{6178., 11 108.}	28.5972	0.105415	newly 3 weeks cumul	30 207.	28 030.6	{22 417., 33 010.}	11.9835	0.0589579	newly 5 weeks spec	2743.	5790.63	{3596., 9538.}	111.148	0.29577	newly 5 weeks cumul	37 399.	40 943.2	{32 405., 50 009.}	16.1525	0.0656662	newly 53 weeks cumul	41 988.	58 828.	{42 114., 82 091.}	42.2865	0.143717
26	newly 3 weeks spec	7069.	5794.55	{3681., 8820.}	28.9733	0.152518	newly 3 weeks cumul	23 874.	20 865.9	{14 656., 29 180.}	22.1328	0.111622	newly 5 weeks spec	4701.	3744.08	{2178., 6014.}	37.2277	0.197102	newly 5 weeks cumul	34 401.	29 314.	{19 718., 42 640.}	25.1876	0.129351	newly 51 weeks cumul	50 936.	41 368.	{26 157., 62 318.}	31.0416	0.162881
27	newly 3 weeks spec	6335.	4108.7	{2196., 6070.}	44.4081	0.26669	newly 3 weeks cumul	22 631.	16 122.8	{9095., 23 601.}	36.44	0.20969	newly 5 weeks spec	3937.	2290.45	{1131., 3306.}	51.7843	0.328618	newly 5 weeks cumul	31 837.	21 502.1	{11 738., 31 564.}	40.5215	0.238778	newly 52 weeks cumul	45 079.	27 566.5	{14 604., 40 639.}	46.0692	0.284448
28	newly 3 weeks spec	7243.	5124.26	{2903., 8086.}	40.7072	0.228773	newly 3 weeks cumul	27 640.	21 334.8	{13 249., 32 046.}	31.0978	0.166976	newly 5 weeks spec	4186.	2551.84	{1311., 3762.}	54.1158	0.324511	newly 5 weeks cumul	37 414.	27 523.6	{16 543., 41 857.}	35.158	0.194002	newly 56 weeks cumul	48 001.	32 782.3	{18 973., 49 489.}	40.6074	0.231872
29	newly 3 weeks spec	6769.	7278.16	{5021., 9300.}	21.0334	0.0875558	newly 3 weeks cumul	23 489.	24 107.5	{18 653., 28 430.}	14.1834	0.0624106	newly 5 weeks spec	4398.	5191.22	{3127., 7448.}	33.2661	0.124537	newly 5 weeks cumul	33 395.	35 556.1	{26 088., 43 293.}	17.818	0.0748183	newly 50 weeks cumul	46 315.	54 573.7	{36 365., 73 079.}	28.2245	0.107024
30	newly 3 weeks spec	8192.	5920.7	{3054., 10 826.}	42.3391	0.241557	newly 3 weeks cumul	34 290.	26 745.9	{16 084., 42 674.}	31.237	0.170146	newly 5 weeks spec	3775.	2514.25	{1049., 4255.}	58.0381	0.337477	newly 5 weeks cumul	43 617.	33 179.4	{18 859., 55 983.}	34.6004	0.192076	newly 59 weeks cumul	50 418.	37 078.8	{20 362., 62 333.}	38.1991	0.214794
31	newly 3 weeks spec	7625.	11 492.8	{6853., 19 017.}	54.1453	0.164417	newly 3 weeks cumul	37 142.	42 665.8	{33 750., 52 075.}	18.1155	0.0700711	newly 5 weeks spec	2494.	5026.42	{2434., 9603.}	103.082	0.243614	newly 5 weeks cumul	43 977.	55 610.	{40 808., 71 837.}	29.069	0.104315	newly 60 weeks cumul	46 875.	62 865.9	{44 222., 87 505.}	36.3251	0.123803
32	newly 3 weeks spec	6240.	7365.73	{4800., 10 296.}	33.4933	0.122298	newly 3 weeks cumul	27 473.	30 243.8	{22 477., 39 725.}	22.6315	0.0901672	newly 5 weeks spec	2353.	3433.2	{2072., 4854.}	51.6249	0.152008	newly 5 weeks cumul	33 807.	38 775.1	{27 609., 51 345.}	26.6753	0.101428	newly 57 weeks cumul	37 286.	45 198.3	{31 408., 60 324.}	30.7848	0.110334
33	newly 3 weeks spec	8467.	7119.5	{4003., 10 709.}	30.3617	0.158373	newly 3 weeks cumul	28 949.	26 517.7	{17 071., 35 963.}	21.4114	0.104698	newly 5 weeks spec	5209.	4024.71	{2101., 6298.}	39.4389	0.214897	newly 5 weeks cumul	40 750.	35 969.6	{22 084., 51 250.}	25.2775	0.127082	newly 55 weeks cumul	56 254.	45 797.4	{26 808., 66 640.}	29.5611	0.157031
34	newly 3 weeks spec	6053.	6560.85	{4245., 9240.}	26.5064	0.110084	newly 3 weeks cumul	19 976.	22 370.4	{15 950., 27 790.}	21.8093	0.0883039	newly 5 weeks spec	4286.	4502.05	{2527., 6791.}	32.7761	0.139827	newly 5 weeks cumul	29 425.	32 396.5	{21 662., 43 141.}	24.1066	0.0987525	newly 50 weeks cumul	43 351.	48 149.7	{29 685., 67 687.}	28.8713	0.116603
35	newly 3 weeks spec	8823.	6731.03	{3551., 11 090.}	32.973	0.185166	newly 3 weeks cumul	31 843.	25 438.7	{14 907., 36 705.}	25.3449	0.142741	newly 5 weeks spec	5272.	3862.12	{1696., 6899.}	43.2201	0.247381	newly 5 weeks cumul	44 053.	34 432.	{19 501., 51 804.}	28.1182	0.155317	newly 55 weeks cumul	57 367.	44 182.3	{23 030., 69 045.}	32.5416	0.181103
36	newly 3 weeks spec	6496.	3691.83	{1829., 6306.}	49.2515	0.317146	newly 3 weeks cumul	23 327.	14 654.2	{7773., 24 486.}	42.2559	0.259386	newly 5 weeks spec	4189.	2062.58	{991., 3532.}	57.1634	0.39399	newly 5 weeks cumul	32 804.	19 498.7	{10 070., 32 758.}	45.7533	0.288386	newly 53 weeks cumul	44 915.	24 925.7	{12 567., 42 392.}	49.8286	0.323732
37	newly 3 weeks spec	6929.	7885.77	{4367., 14 263.}	40.8782	0.158196	newly 3 weeks cumul	31 730.	33 082.7	{22 234., 48 062.}	25.6908	0.11013	newly 5 weeks spec	2540.	3241.68	{1590., 6599.}	52.603	0.179588	newly 5 weeks cumul	38 553.	41 498.	{26 584., 64 877.}	29.9302	0.1235	newly 58 weeks cumul	42 395.	46 506.6	{29 041., 75 206.}	32.2941	0.13025
38	newly 3 weeks spec	5419.	10 308.4	{6898., 15 113.}	91.5871	0.267528	newly 3 weeks cumul	32 641.	36 481.4	{27 193., 44 190.}	22.7707	0.102416	newly 5 weeks spec	1229.	5917.22	{3039., 9900.}	381.466	0.632096	newly 5 weeks cumul	36 650.	50 370.2	{37 541., 64 200.}	39.4411	0.138903	newly 58 weeks cumul	37 768.	63 204.5	{43 670., 85 376.}	67.4254	0.210692
39	newly 3 weeks spec	5970.	7460.82	{4829., 10 603.}	35.9758	0.154038	newly 3 weeks cumul	21 972.	24 874.8	{18 402., 30 477.}	24.3438	0.125201	newly 5 weeks spec	3152.	5255.98	{2824., 9448.}	73.3796	0.222725	newly 5 weeks cumul	29 579.	36 508.6	{24 980., 48 024.}	32.3786	0.139988	newly 52 weeks cumul	36 448.	55 811.3	{34 699., 84 290.}	56.2107	0.178478
40	newly 3 weeks spec	6214.	5655.32	{2950., 9416.}	33.2641	0.161618	newly 3 weeks cumul	22 734.	21 455.1	{12 400., 31 782.}	24.5347	0.11868	newly 5 weeks spec	3630.	3207.55	{1492., 5261.}	43.0152	0.207317	newly 5 weeks cumul	31 216.	28 976.3	{15 903., 45 624.}	28.5569	0.138035	newly 53 weeks cumul	41 146.	37 073.8	{19 357., 59 077.}	32.6917	0.159421
41	newly 3 weeks spec	5376.	7347.86	{4975., 10 428.}	40.6124	0.139035	newly 3 weeks cumul	21 206.	26 706.3	{19 051., 34 864.}	30.9975	0.114857	newly 5 weeks spec	2830.	4542.52	{2496., 7142.}	64.8227	0.183917	newly 5 weeks cumul	27 896.	37 116.3	{26 271., 49 582.}	35.8846	0.126916	newly 54 weeks cumul	33 607.	49 480.9	{32 544., 70 943.}	49.6428	0.160391
42	newly 3 weeks spec	4668.	5300.06	{2593., 9713.}	46.6774	0.177423	newly 3 weeks cumul	24 350.	24 198.1	{13 971., 40 794.}	33.4767	0.147975	newly 5 weeks spec	1451.	2168.39	{997., 4001.}	68.2355	0.187447	newly 5 weeks cumul	28 470.	29 813.1	{16 547., 51 364.}	37.1927	0.15519	newly 58 weeks cumul	30 111.	33 172.7	{18 019., 57 617.}	40.0318	0.157532
43	newly 3 weeks spec	8069.	7740.04	{5153., 10 083.}	19.5583	0.0931868	newly 3 weeks cumul	29 202.	24 954.8	{18 092., 29 663.}	15.0506	0.0811648	newly 5 weeks spec	4750.	5763.87	{3187., 8904.}	37.8146	0.141993	newly 5 weeks cumul	40 208.	37 518.2	{26 747., 45 977.}	15.7104	0.0766426	newly 51 weeks cumul	53 458.	59 230.5	{37 362., 81 537.}	26.6762	0.108845
44	newly 3 weeks spec	6354.	5494.6	{2915., 8611.}	33.3971	0.167553	newly 3 weeks cumul	27 932.	24 008.2	{13 841., 35 624.}	27.8181	0.141881	newly 5 weeks spec	2650.	2455.52	{1224., 3782.}	36.6949	0.171956	newly 5 weeks cumul	34 711.	30 173.3	{17 048., 45 057.}	29.3947	0.147907	newly 57 weeks cumul	39 102.	34 682.6	{19 333., 52 131.}	30.4636	0.150236
45	newly 3 weeks spec	4797.	8776.43	{5667., 13 084.}	82.9567	0.242894	newly 3 weeks cumul	26 104.	32 113.7	{23 640., 39 687.}	27.9172	0.10474	newly 5 weeks spec	1420.	4899.67	{2685., 8296.}	245.047	0.499709	newly 5 weeks cumul	30 202.	43 686.6	{30 695., 58 979.}	45.3536	0.151767	newly 56 weeks cumul	31 840.	54 716.1	{36 635., 77 908.}	71.8471	0.218594
46	newly 3 weeks spec	8356.	9504.29	{6290., 13 318.}	27.5142	0.108336	newly 3 weeks cumul	33 903.	32 759.4	{25 750., 38 885.}	12.9422	0.0597849	newly 5 weeks spec	3539.	5597.53	{3086., 9602.}	64.2937	0.185788	newly 5 weeks cumul	42 756.	45 850.1	{33 962., 57 845.}	18.8446	0.0785366	newly 56 weeks cumul	48 435.	58 559.1	{40 342., 81 780.}	29.1862	0.107905
47	newly 3 weeks spec	6682.	8953.35	{5466., 13 397.}	42.028	0.142474	newly 3 weeks cumul	30 081.	35 316.6	{25 772., 45 121.}	24.1281	0.0934305	newly 5 weeks spec	2681.	4161.65	{2172., 6348.}	62.7134	0.182471	newly 5 weeks cumul	37 114.	45 724.1	{31 462., 62 025.}	30.1553	0.111649	newly 58 weeks cumul	40 818.	52 980.7	{35 231., 73 157.}	36.1265	0.127413
48	newly 3 weeks spec	8702.	10 040.8	{6746., 14 312.}	28.1785	0.108075	newly 3 weeks cumul	37 680.	37 396.4	{29 296., 43 703.}	12.8416	0.0575982	newly 5 weeks spec	3585.	4940.49	{2927., 7826.}	46.3308	0.149993	newly 5 weeks cumul	46 811.	49 559.8	{36 706., 63 250.}	18.3387	0.0767684	newly 58 weeks cumul	52 174.	58 977.1	{42 004., 78 496.}	23.2558	0.0910908
49	newly 3 weeks spec	4127.	8025.29	{4206., 13 107.}	96.1459	0.251637	newly 3 weeks cumul	27 465.	36 808.9	{23 705., 52 478.}	39.8402	0.137248	newly 5 weeks spec	921.	2791.15	{1317., 4653.}	203.056	0.415444	newly 5 weeks cumul	30 359.	44 438.8	{27 319., 66 286.}	50.611	0.162725	newly 60 weeks cumul	31 206.	47 998.4	{28 950., 72 214.}	57.2668	0.177768
50	newly 3 weeks spec	6476.	7024.73	{4534., 10 094.}	26.5777	0.110434	newly 3 weeks cumul	23 838.	24 236.1	{17 287., 30 416.}	18.1192	0.0837559	newly 5 weeks spec	3992.	4702.13	{2763., 7491.}	36.1405	0.136081	newly 5 weeks cumul	32 964.	34 760.9	{23 230., 47 068.}	21.9616	0.0935763	newly 51 weeks cumul	44 879.	50 767.3	{32 958., 73 356.}	28.7127	0.112671
